# The interplay of cellular senescence and reprogramming shapes the biological landscape of aging and cancer revealing novel therapeutic avenues

**DOI:** 10.3389/fcell.2025.1593096

**Published:** 2025-04-28

**Authors:** Fuan Ding, Ying Yu, Jiangqi Zhao, Shibo Wei, Yan Zhang, Jung Ho Han, Zhuo Li, Hong-Bo Jiang, Dongryeol Ryu, Minkyoung Cho, Sung-Jin Bae, Wonyoung Park, Ki-Tae Ha, Bo Gao

**Affiliations:** ^1^ Department of Vascular Surgery, The Second Hospital of Jilin University, Changchun, China; ^2^ Department of Surgery, Changchun University of Chinese Medicine, Changchun, China; ^3^ Department of Dermatology, The Second Hospital of Jilin University, Changchun, China; ^4^ Department of Biomedical Science and Engineering, Gwangju Institute of Science and Technology, Gwangju, Republic of Korea; ^5^ Korean Medicine Application Center, Korea Institute of Oriental Medicine, Daegu, Republic of Korea; ^6^ Department of Nephrology, Shandong Provincial Hospital Affiliated to Shandong First Medical University, Jinan, Shandong, China; ^7^ Department of Dermatology, Qingdao Women and Children’s Hospital, Qingdao University, Qingdao, Shandong, China; ^8^ Department of Parasitology and Tropical Medicine, and Institute of Health Sciences, Gyeongsang National University College of Medicine, Jinju, Republic of Korea; ^9^ Department of Molecular Biology and Immunology, Kosin University College of Medicine, Busan, Republic of Korea; ^10^ Department of Korean Medical Science, School of Korean Medicine, Pusan National University, Yangsan, Gyeongsangnam-do, Republic of Korea; ^11^ Research Institute for Korean Medicine, Pusan National University, Yangsan, Gyeongsangnam-do, Republic of Korea

**Keywords:** aging, cancer, cellular senescence, reprogramming, tumor progression

## Abstract

Cellular senescence and cellular reprogramming represent two fundamentally intertwined processes that profoundly influence aging and cancer. This paper explores how the permanent cell-cycle arrest of senescent cells and the identity-resetting capacity of reprogramming jointly shape biological outcomes in later life and tumor development. We synthesize recent findings to show that senescent cells, while halting the proliferation of damaged cells, can paradoxically promote tissue dysfunction and malignancy via their secretory phenotype. Conversely, induced reprogramming of somatic cells—exemplified by Yamanaka factors—resets cellular age and epigenetic marks, offering a potential to rejuvenate aged cells. Key findings highlight shared mechanisms (e.g., DNA damage responses and epigenetic remodeling) and bidirectional crosstalk between these processes: senescence signals can facilitate neighboring cell plasticity, whereas reprogramming attempts can trigger intrinsic senescence programs as a barrier. In aging tissues, transient (partial) reprogramming has been shown to erase senescence markers and restore cell function without inducing tumorigenesis, underlining a novel strategy to combat age-related degeneration. In cancer, we discuss how therapy-induced senescence of tumor cells may induce stem-cell-like traits in some cells and drive relapse, revealing a delicate balance between tumor suppression and tumor promotion. Understanding the interplay between senescence and reprogramming is crucial for developing innovative therapies. By targeting the senescence–reprogramming axis–for instance, via senolytic drugs, SASP inhibitors, or safe reprogramming techniques–there is significant therapeutic potential to ameliorate aging-related diseases and improve cancer treatment. Our findings underscore that carefully modulating cellular senescence and rejuvenation processes could pave the way for novel regenerative and anti-cancer strategies.

## 1 Introduction

Cellular senescence and cellular reprogramming, although seemingly opposing processes, profoundly influence aging and cancer through their interconnected roles in cellular fate determination (Menendez and Alarcón, 2017; Calcinotto et al., 2019). Senescence is characterized by irreversible cell-cycle arrest and secretion of inflammatory mediators (senescence-associated secretory phenotype, SASP), serving initially as a tumor-suppressive mechanism (Loaiza and Demaria, 2016; Roger et al., 2021). However, chronic accumulation of senescent cells contributes significantly to aging and age-related diseases by creating a pro-inflammatory, pro-tumorigenic environment (Fülöp et al., 2016; Blasiak, 2020). Conversely, cellular reprogramming—achieved by introducing transcription factors such as Octamer-binding transcription factor 4 (OCT4), SRY-box transcription factor 2 (SOX2), Kruppel-like factor 4 (KLF4), and MYC proto-oncogene, bHLH transcription factor (MYC) (collectively OSKM)—resets cellular aging markers and rejuvenates aged cells by restoring proliferative and regenerative capacity (Huyghe et al., 2024). Recent research reveals a complex crosstalk between these seemingly opposing processes (Schmeer et al., 2019; Chiche et al., 2020). Senescence checkpoints can restrict reprogramming efficiency, while SASP components can paradoxically enhance cellular plasticity, influencing regeneration or tumor progression based on the context (Birch and Gil, 2020; Zhang et al., 2024).

This review explores the dynamic interplay between senescence and reprogramming, emphasizing their shared molecular pathways and reciprocal influence on aging and cancer. Therapeutic strategies such as senolytics (eliminating senescent cells), senomorphics (modulating SASP), and controlled partial reprogramming are highlighted as promising approaches to mitigate aging effects and prevent malignancies. A better understanding of the senescence-reprogramming axis offers novel avenues for interventions that could significantly improve healthspan and cancer outcomes.

## 2 Hallmarks of cancer and aging

Since Hanahan and Weinberg first introduced the concept of the hallmarks of cancer in 2000, researchers have identified key biological capabilities that drive tumor growth and progression. Initially, six capabilities were outlined, including evasion of apoptosis, self-sufficiency in growth signals, resistance to anti-growth signals, sustained angiogenesis, limitless replicative potential, and the ability to invade tissues and metastasize (Hanahan and Weinberg, 2000). This framework was later expanded in 2011 to include emerging hallmarks such as deregulated cellular energetics and avoiding immune destruction, along with enabling characteristics like genome instability and tumor-promoting inflammation (Hanahan and Weinberg, 2011). Notably, the investigation into how tumor cells circumvent immune surveillance became a major research focus, eventually leading to the development of immune checkpoint inhibitors, a breakthrough that revolutionized cancer therapy (Zhang and Zhang, 2020). This paradigm shift was internationally recognized when the 2018 Nobel Prize in Physiology or Medicine was awarded to James P. Allison and Tasuku Honjo for their pioneering contributions to immunotherapy (Gilman, 2019). In 2022, Hanahan further refined the model by adding new dimensions that reflect advances in cancer research, including unlocking phenotypic plasticity, recognizing the role of nonmutational epigenetic reprogramming, understanding the impact of polymorphic microbiomes, and emphasizing the accumulation of senescent cells as a hallmark of cancer (Hanahan, 2022). These new dimensions highlight how dynamic alterations in cellular identity play a central role in both the initiation and progression of cancer. Specifically, unlocking phenotypic plasticity enables cells to adapt to changing microenvironments by shifting their characteristics, which can lead to treatment resistance and support tumor growth (Diazzi et al., 2023; Bhat et al., 2024). Similarly, nonmutational epigenetic reprogramming modifies gene expression without altering the DNA sequence, facilitating the transformation of normal cells into malignant ones while also contributing to the buildup of senescent cells—cells that, despite having lost the ability to proliferate, can secrete inflammatory factors that further promote tumor progression and impact aging (Davalos et al., 2010; López-Otín et al., 2023). Cellular senescence, once considered solely a tumor-suppressive mechanism, is now increasingly recognized as a double-edged sword—a hallmark that bridges cancer and aging by exerting both anti-proliferative and pro-tumorigenic effects depending on context (Xiao et al., 2023). Collectively, these insights emphasize that systemic and environmental factors are intricately linked with cancer development and the aging process, underscoring the complexity of these intertwined biological phenomena (López-Otín et al., 2023).

Similarly, the “hallmarks of aging” were articulated by López-Otín et al., in 2013, defining nine key processes that drive aging: genomic instability, telomere attrition, epigenetic alterations, loss of proteostasis, deregulated nutrient sensing, mitochondrial dysfunction, cellular senescence, stem cell exhaustion, and altered intercellular communication (López-Otín et al., 2013). These hallmarks provide insight into the molecular and cellular processes underlying the aging process and age-related diseases, establishing a comprehensive framework analogous to that of cancer research. Recent advancements have further emphasized the role of the gut microbiome, chronic inflammation, and metabolic shifts in aging, broadening the understanding of how systemic factors influence aging-related decline (Ling et al., 2022).

**FIGURE 1:**
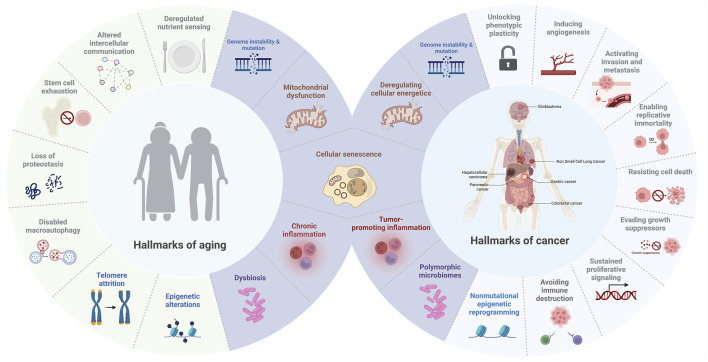
Hallmarks of aging and cancer. This figure illustrates the overlapping and distinct biological processes that connect aging and cancer, highlighting common hallmarks such as cellular senescence, mitochondrial dysfunction, genome instability and mutation, inflammation, and dysbiosis (in purple).

While cancer, characterized by unchecked cellular growth, and aging, marked by a decline in cellular function, may appear diametrically opposed, they share overlapping biological hallmarks that underscore their interconnection (Figure 1) (Anisimov et al., 2009). Among these, cellular senescence emerges as a particularly significant shared hallmark of both cancer and aging. In the context of cancer, senescence acts as a barrier to malignant transformation by inducing stable growth arrest in damaged cells (Schmitt et al., 2022). However, senescent cells can also acquire SASP, which promotes inflammation, tissue remodeling, and even tumor progression in certain microenvironments (Reynolds et al., 2024). Similarly, in aging, the accumulation of senescent cells contributes to tissue dysfunction and chronic inflammation, and is now recognized as a major driver of age-related pathologies such as osteoarthritis, pulmonary fibrosis, and metabolic diseases (Kaur and Farr, 2020). Importantly, emerging evidence now highlights the role of cell reprogramming in this context, revealing that alterations in cellular identity not only influence regenerative capacity but also intersect with senescence pathways to drive oncogenesis or mitigate tumor progression (Schmeer et al., 2019; Baechle et al., 2023). This reprogramming-senescence axis exemplifies the complex interplay between mechanisms that govern tissue repair, aging, and cancer, a topic we will explore further in the next section.

## 3 Crosstalk between senescence and cellular reprogramming

**FIGURE 2:**
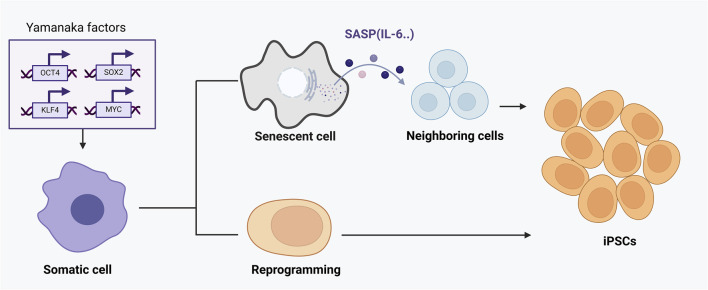
The dual roles of senescence and cellular reprogramming in aging and tissue regeneration. This figure demonstrates the complex interaction between cellular reprogramming and senescence mediated by OSKM factors (Oct4, Sox2, Klf4, c-Myc). Upon OSKM induction, a subset of cells undergoes direct and successful reprogramming into induced pluripotent stem cells (iPSCs), rejuvenating cellular function. However, other cells fail to fully reprogram and instead enter a senescent state, characterized by stable cell-cycle arrest and secretion of the senescence-associated secretory phenotype (SASP). SASP components, particularly cytokines like IL-6, act in a paracrine manner to enhance reprogramming efficiency in adjacent cells. Thus, cellular senescence, through SASP, paradoxically provides a supportive niche that boosts reprogramming and tissue regeneration. The outcome of this interplay depends critically on the balance between reprogramming success and senescence induction, influencing tissue repair, aging progression, and regenerative capacity.

Accumulating studies show that senescence and reprogramming are mechanistically intertwined rather than mutually exclusive (Drapela et al., 2022). Induction of reprogramming factors *in vivo* often triggers senescence in a subset of cells, while successfully reprogrammed neighboring cells emerge in parallel (Mosteiro et al., 2018). In a landmark mouse model, the activation of OSKM (Oct4, Sox2, Klf4, c-Myc) in adult tissues led some cells to undergo senescence and secrete SASP factors, which paradoxically promoted the reprogramming of nearby cells (Rattanavirotkul et al., 2021). Specifically, senescent cells released interleukin-6 (IL-6) and other cytokines that acted in a paracrine fashion enhanced the efficiency of OSKM-mediated conversion of surrounding cells to induced pluripotent stem cells (iPSCs) (Mosteiro et al., 2018) (Figure 2). In this way, cellular senescence provides a supportive niche for reprogramming: the damage-induced senescent cells halt their proliferation but produce signals that increase the plasticity of other cells (Ji et al., 2023). Notably, if the senescence program is genetically disabled (e.g., knocking out p16^Ink4a/Arf^ in that model), reprogramming efficiency *in vivo* drops dramatically, underscoring that the senescence-associated secretome is a crucial driver of cell-fate plasticity (D’Arcangelo et al., 2017). Thus, senescence can facilitate reprogramming through SASP-mediated crosstalk, linking these processes as cooperative regulators of cell fate under stress conditions (D’Arcangelo et al., 2017).

Conversely, cellular reprogramming influences senescence (Chen and Skutella, 2022). Reprogramming somatic cells to pluripotency resets numerous aging markers and can effectively “rejuvenate” cells that were previously senescent or biologically old (Alle et al., 2021). For example, human fibroblasts from elderly donors (even centenarians) regain longer telomeres, youthful gene expression profiles, and proliferative vigor after reprogramming to iPSCs (Blake, 2025). The reprogrammed cells lose senescence hallmarks and acquire the characteristics of embryonic-like cells, indicating that the reprogramming process can erase cellular aging features and senescent phenotypes (Studer et al., 2015). This “reset” underscores an intrinsic connection between the epigenetic state of a cell (which is reset by reprogramming) and the senescence program (which is driven by an aging epigenome) (Ashapkin et al., 2017). However, the interplay is context-dependent: while full reprogramming wipes senescent features, partial reprogramming or aborted reprogramming attempts can induce senescence (Niemann, 2016; Galkin et al., 2019). Cells that undergo oncogenic stress or incomplete reprogramming often activate p53/p21 and enter senescence as a safeguard against tumorigenic transformation (Schmitt et al., 2022). Thus, reprogramming can both abolish senescence (when successful and complete) and provoke senescence (when perceived as cellular stress or when only partially successful) (Mosteiro et al., 2016). The net outcome depends on the balance and timing of these processes, highlighting a delicate co-regulation of cell fate–senescence limits immediate proliferation but can create a pro-reprogramming milieu, whereas reprogramming can rejuvenate cells but, if misinitiated, triggers senescence as a quality control (Geenen et al., 2013; Kim, 2015).

## 4 Interplay in tissue homeostasis, aging, and regeneration

The crosstalk between senescence and reprogramming is critical for tissue homeostasis and organismal aging. Transient induction of senescence is now recognized as a normal component of tissue remodeling and regeneration (Ritschka et al., 2017). During wound healing and embryonic development, waves of senescent cells secrete growth factors and mitogens that stimulate stem/progenitor cells and promote tissue repair (Farooq et al., 2021). Short-term exposure of neighboring cells to the SASP can enhance their plasticity and even increase the efficiency of reprogramming factors *in vivo* (Giroud et al., 2023). In mouse models, experimentally inducing a temporary burst of senescent cells in damaged tissues (e.g., via an oncogene or irradiation) has been shown to improve regeneration of muscle, skin, liver, and heart by encouraging cell dedifferentiation and proliferation of progenitors (Forbes and Rosenthal, 2014; Schafer et al., 2018). In one study, transient SASP signals (including IL-6, IL-8, and growth factors) boosted reprogramming efficiency and tissue repair, but prolonged SASP exposure had the opposite effect–prolonged senescence was sensed as aberrant, activating barriers that blocked excessive cell plasticity (Rhinn et al., 2019; Cuollo et al., 2020). These findings illustrate a yin-yang relationship: an acute senescence response can set the stage for reprogramming and regeneration, aiding tissue homeostasis after injury, whereas chronic senescence (accumulation of senescent cells with age) impairs regeneration and contributes to degenerative changes (Wilkinson and Hardman, 2022).

During aging, an imbalance in this interplay is evident. Aged tissues accumulate senescent cells that secrete pro-inflammatory SASP factors long-term, disrupting stem cell niches and tissue integrity (Khalil et al., 2023; Li et al., 2023). At the same time, the ability to reprogram or rejuvenate cells declines with age–partly due to these same senescence-related changes (Turinetto et al., 2016; Zhou et al., 2020). Chronic SASP signaling in aged tissues can enforce nearby cells into dysfunction or aberrant differentiation, limiting the regenerative capacity (Ovadya and Krizhanovsky, 2014). Indeed, studies have found that clearing senescent cells in progeroid or naturally aged mice improves tissue function and can even extend lifespan (Naylor et al., 2013). Senescent cells actively drive aging phenotypes: they cause local inflammation, matrix degradation, and stem cell inhibition, leading to frailty and organ decline (Childs et al., 2015). Removal of these cells with senolytic strategies enhanced tissue rejuvenation and physical function in old mice (Xu et al., 2018). This suggests that the age-related decline in tissue homeostasis is partly due to excessive senescence tipping the balance away from regenerative reprogramming (Yun, 2015). Supporting this, the induction of a youthful gene program via controlled reprogramming has shown promise in counteracting aging (Yun, 2015; Roux et al., 2022). In a groundbreaking experiment, cyclic expression of Yamanaka factors (OSKM) in middle-aged mice was able to reverse multiple hallmarks of aging in cells and improve tissue function without causing tumors (Takahashi and Yamanaka, 2016). Short-term, intermittent reprogramming in a progeroid mouse model ameliorated fibrosis, restored organ function, and even extended the animals’ lifespan (Culig et al., 2023). Similarly, partial reprogramming improved muscle regeneration and metabolic recovery in older wild-type mice, highlighting that aged cells still harbor latent regenerative potential that can be unlocked (Mozzetta et al., 2024). Thus, aging can be viewed as a shift in the senescence–reprogramming equilibrium: accumulated senescence and epigenetic noise suppress regenerative reprogramming, but strategic interventions can restore some youthful reprogramming capacity, leading to tissue rejuvenation (Rando and Chang, 2012; Zhang et al., 2020).

Recent findings underscore how senescence influences reprogramming efficiency in regeneration. For instance, senescent cell secretions have been shown to activate regenerative pathways in tissues (Ritschka et al., 2017). In the heart, transient p53-mediated “regenerative senescence” after injury was found to spur cardiomyocyte proliferation and repair, partly via SASP growth factors (Zhai and Sadoshima, 2024). In skeletal muscle injury, a subset of stromal cells undergo transient senescence and drive muscle stem cell expansion for repair, an effect lost if those senescent cells are removed too early (Sousa-Victor et al., 2022). Moreover, the interplay works in the opposite direction during regeneration: introducing reprogramming factors or exposing aged cells to a youthful environment can reduce markers of senescence (Rando and Chang, 2012). Heterochronic parabiosis and cell fusion experiments suggest systemic factors in young circulation can rejuvenate old cells, essentially reprogramming certain epigenetic aspects and lowering the senescent burden in tissues (Schmeer et al., 2019). While the exact molecules are still being identified, it is likely that pro-youthful factors (e.g., growth differentiation factor 11 and others) counteract senescence-induced epigenetic changes (Liu et al., 2022). Collectively, these observations indicate that maintaining tissue homeostasis and regenerative potential relies on a finely tuned crosstalk: a transient senescence program can kick-start repair by encouraging cell plasticity, but sustained senescence will impede regeneration (Wilkinson and Hardman, 2020). Therapeutically tipping this balance–for example, by removing chronically senescent cells or by providing rejuvenation factors–is a promising route to enhance regeneration in aged or damaged tissues (Oh et al., 2014).

## 5 Senescence as a pro-tumorigenic factor in aging tissues

Cellular senescence is a tightly regulated process initiated by various intrinsic and extrinsic stressors, among which DNA damage is one of the most potent inducers (Kumari and Jat, 2021). The DNA damage response (DDR), through activation of ATM/ATR kinases, leads to stabilization of the tumor suppressor p53, which in turn promotes the expression of cell cycle inhibitors such as p21^cip1^ (Mijit et al., 2020). This pathway collaborates with the p16^INK4a–Rb^ axis to enforce a stable growth arrest characteristic of senescence (Kumari and Jat, 2021). The p53–p21 and p16–Rb pathways are thus central to both the induction and long-term maintenance of senescence, acting as a robust barrier against malignant transformation (Kumari and Jat, 2021). However, when components of these pathways are mutated or silenced—such as p53 loss-of-function—cells can bypass senescence, leading to uncontrolled proliferation and tumor development (Dimri, 2005). This loss of senescence stability is a key step in tumorigenesis and contributes to the emergence of aggressive, therapy-resistant cancer phenotypes (Schmitt et al., 2022).

**FIGURE 3:**
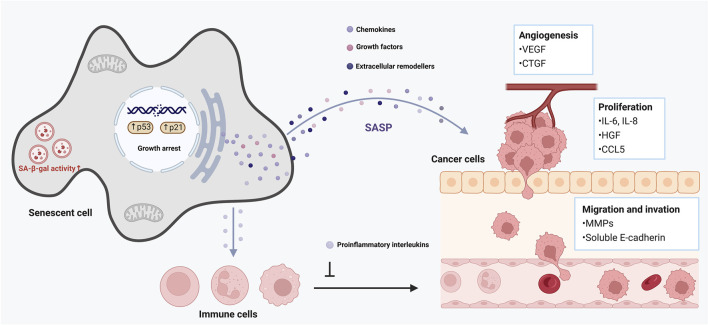
Role of cellular senescence in tumor promotion. This figure illustrates the complex role of cellular senescence in aging and cancer development. Senescent cells remain metabolically active and secrete various pro-inflammatory cytokines, chemokines, growth factors, and proteases, collectively known as the senescence-associated secretory phenotype (SASP). The SASP can reinforce growth arrest, thereby strengthening the halt in cell proliferation, and recruit immune cells to clear senescent cells. However, it can also promote a pro-tumorigenic environment by inducing chronic inflammation, disrupting tissue architecture, and stimulating the proliferation of nearby pre-malignant cells.

Cellular senescence is a double-edged sword in cancer (Xiao et al., 2023). On one hand, senescence is a critical tumor-suppressive mechanism: damaged cells (e.g., with oncogenic mutations) are forced into senescence (oncogene-induced senescence, OIS), halting their progression to malignancy (Hinds and Pietruska, 2017). On the other hand, senescent cells (especially in the tumor microenvironment) can promote cancer development via their secretory phenotype (Takasugi et al., 2022). The interplay between senescence and cellular plasticity (a form of reprogramming in tumors) plays a decisive role in tumor initiation and progression (Vernot, 2020). Early in tumorigenesis, induction of senescence in premalignant cells (mediated by p16^INK4a^, p53, etc.) creates a barrier that nascent cancer cells must overcome to form a tumor (Rajaraman et al., 2006). Cells that manage to escape or bypass senescence often do so by acquiring stem-like or progenitor-like features–effectively a reprogramming event (Trosko, 2009). Intriguingly, recent work indicates that tumor cells escaping senescence exhibit increased cellular plasticity and stemness (De Blander et al., 2021). In other words, if a cancer cell evades the senescence arrest (for example, by losing p53 function or upregulating telomerase), it does not just proliferate–it may also de-differentiate toward a more aggressive, stem-like state (Steinbichler et al., 2018). This conversion is driven in part by the senescence/SASP environment: SASP factors like IL-6 and IL-8 from nearby senescent stromal cells can induce epithelial–mesenchymal transition (EMT) and other reprogramming-like changes in incipient cancer cells, endowing them with invasive, metastatic capabilities (Ortiz-Montero et al., 2017) (Figure 3). Thus, the senescence program initially restrains tumor growth, but the paracrine effects of senescent cells can paradoxically fuel tumor progression by altering the phenotype of surrounding cells (Campisi, 2013).

In established tumors, a dynamic interplay persists. Within many cancers, subpopulations of cells can enter a state of stable arrest similar to senescence (often in response to stress or therapy), while other subpopulations maintain proliferation (Gewirtz et al., 2008). The SASP from senescent tumor cells or senescent stromal fibroblasts can support the growth of non-senescent cancer cells by providing growth factors, pro-angiogenic signals, and immunosuppressive cytokines (Ye et al., 2023). This creates a heterogenous tumor ecosystem where senescent cells serve as “fertilizer” for their more malignant neighbors (Riviere-Cazaux et al., 2023). For example, in breast cancer models, senescent fibroblasts secreting IL-6/IL-8 significantly enhanced the invasiveness and stemness of nearby carcinoma cells (Ortiz-Montero et al., 2017). The reprogramming toward stem-like phenotypes in cancer (often called cancer cell plasticity) is influenced by these senescence-associated signals (Poli et al., 2018). Cancer cells can toggle between more differentiated states and more primitive, therapy-resistant states; SASP factors push them toward the latter (Walcher et al., 2020). Indeed, chronic inflammation and SASP in the tumor microenvironment have been shown to induce a spectrum of hybrid epithelial/mesenchymal phenotypes and bolster the cancer stem cell pool (Chambers et al., 2021). This plasticity contributes to drug resistance and metastasis, as stem-like cancer cells are better equipped to survive chemotherapy and colonize new niches (Plaks et al., 2015).

Senescence and reprogramming also intersect during cancer therapy. Many frontline treatments (chemotherapy, radiation, some targeted drugs) do not kill all tumor cells; instead, a fraction of cancer cells survive by entering a therapy-induced senescence state (Chakrabarty et al., 2021; Xu et al., 2024). These senescent tumor cells are growth-arrested but metabolically active and often secrete abundant SASP factors (Mijit et al., 2020). If left in place, therapy-induced senescent cells can promote relapse: they induce inflammation, stimulate angiogenesis, and can even eventually re-enter the cell cycle or encourage neighboring surviving cells to become more aggressive (Mijit et al., 2020; Schmitt et al., 2022). There is evidence that after certain chemotherapies, the residual senescent cancer cells acquire markers of stemness or mesenchymal identity as they attempt to escape senescence, resembling a partial reprogramming that leads to tumor regeneration (Triana-Martínez et al., 2020; Marzagalli et al., 2021). This realization has spurred interest in eliminating senescent cells in tumors to improve outcomes. Approaches such as senolytic drugs (which specifically kill senescent cells) are being explored to purge therapy-induced senescent tumor cells and thereby prevent cancer recurrence (Wyld et al., 2020). Conversely, researchers are investigating if forcing highly aggressive cancer cells into a terminal senescence or a more differentiated state (a kind of inverse reprogramming) could tame the disease (Trosko, 2009). While inducing permanent senescence in cancer cells is desirable to stop proliferation, any such strategy must contend with the negative effects of SASP. Overall, in cancer biology the senescence–reprogramming interplay creates a vicious cycle: the stress-induced senescence of some cells leads to secretions that can “reprogram” other cells to a stem-like, resistant state, driving heterogeneity and progression (Menon et al., 2020). Breaking this cycle is a key therapeutic challenge.

## 6 Senescence as a pathological driver in age-related degenerative diseases

Senescent cells accumulate with advancing age, and their SASP drives chronic low-grade inflammation and pathological tissue remodeling that contribute to many age (Tominaga, 2015). In idiopathic pulmonary fibrosis, for example, senescent alveolar epithelial cells and fibroblasts secrete SASP factors such as TGF-β1, IL-6, and matrix metalloproteinases that perpetuate fibroblast activation and collagen deposition, leading to progressive scarring of the lung parenchyma (Hernandez-Gonzalez et al., 2021; Lu et al., 2025). Likewise, in metabolic disorders like obesity and type 2 diabetes, senescent cells in adipose tissue (and other metabolic organs) foster chronic “metaflammation” – a state of persistent, sterile inflammation–and disrupt normal tissue homeostasis, exacerbating insulin resistance and pancreatic β-cell dysfunction (Kawai et al., 2021). In osteoarthritis, the late-life degeneration of joints, senescent chondrocytes and synovial fibroblasts accumulate in cartilage and joint tissues and release pro-inflammatory cytokines and matrix-degrading enzymes that erode the extracellular matrix, drive joint inflammation, and impair tissue repair (Sanchez-Lopez et al., 2022). Mechanistically, the SASP creates a pro-inflammatory microenvironment (e.g., via IL-1, IL-6, IL-8) and releases proteases and growth factors that together fuel chronic inflammation and aberrant tissue remodeling, thereby linking cellular senescence to the pathogenesis of fibrosis, metabolic dysfunction, and degenerative diseases (Birch and Gil, 2020; Guo et al., 2022; Yue et al., 2022). These insights have spurred the development of “senotherapeutic” strategies to target senescent cells in aging tissues. Senolytic agents (such as the dasatinib–quercetin combination) selectively induce apoptosis in senescent cells by disabling their survival pathways, thereby reducing the burden of SASP-producing cells and alleviating inflammation (Hickson et al., 2019). Senomorphic compounds, in contrast, aim to modulate or suppress the SASP profile of senescent cells without killing them, blunting the chronic inflammatory signaling and tissue-destructive effects of these cells (Zhang et al., 2023). Notably, preclinical studies in models of pulmonary fibrosis, metabolic syndrome, and osteoarthritis have shown that both senolytic clearance of senescent cells and SASP inhibition can attenuate pathology–for instance, removing senescent cells improves lung function and fibrotic lesions in injured lungs and enhances insulin sensitivity in obese mice (Waters et al., 2018; Boccardi and Mecocci, 2021; Liu et al., 2022). Early-phase clinical trials of senolytics are now underway, and initial results indicate improved tissue function in conditions such as idiopathic pulmonary fibrosis and diabetic kidney disease (Wissler Gerdes et al., 2021). Together, these findings underscore the pathological role of senescent cells in diverse age-related diseases and highlight the therapeutic potential of senolytics and senomorphics as emerging interventions to abrogate SASP-driven chronic inflammation and tissue deterioration.

## 7 Therapeutic implications and interventions

Understanding the interplay between senescence and reprogramming opens exciting therapeutic avenues for both aging and cancer (Zhang et al., 2024). If senescence and reprogramming co-regulate tissue health and disease progression, then interventions can be designed to modulate one or both processes to restore balance (Kane and Sinclair, 2019). Here, we refine the therapeutic implications by discussing strategies that target senescent cells or leverage controlled reprogramming, with the ultimate goal of improving outcomes in aging-related degenerative diseases and cancer.

### 7.1 Senolytics and senomorphics

One strategy is to reduce the burden of senescent cells and their deleterious secretions. Senolytics are drugs that selectively induce death of senescent cells, thereby removing their influence (Kirkland and Tchkonia, 2020) (Table 1). Proof-of-concept studies in mice showed that periodic clearance of senescent cells can rejuvenate tissues, improving physical function and even extending lifespan (Paez-Ribes et al., 2019). For example, the senolytic combination of dasatinib plus quercetin cleared senescent cells in aged mice, leading to increased exercise endurance, improved cardiac function, and delayed osteoporosis (Zhu et al., 2020). In the context of cancer, senolytics are being tested as adjuncts to therapy: by eliminating therapy-induced senescent cells in tumors, they may prevent the pro-tumorigenic SASP effects and reduce recurrence (Piskorz and Cechowska-Pasko, 2022). Early preclinical models have shown that adding senolytics after chemotherapy can decrease inflammation and tumor regrowth (Schmitt et al., 2022). A related approach involves senomorphics (also known as senostatics), which are agents that suppress the SASP or alter senescent cell behavior without killing the cells (Lagoumtzi and Chondrogianni, 2021) (Table 2). The goal with senomorphics is to mitigate the “dark side” of senescent cells (their inflammatory secretome) while retaining any transient beneficial roles (Khalil et al., 2023). Notable examples include Janus kinase/signal transducer and activator of transcription (JAK/STAT) pathway inhibitors, rapamycin, and metformin–all of which have been found to dampen SASP signaling (Xu et al., 2016). Rapamycin and metformin, in fact, are considered geroprotective drugs that extend lifespan in animal models, partly by inhibiting mechanistic target of rapamycin (mTOR)-driven SASP production (rapamycin) or nuclear factor kappa-light-chain-enhancer of activated B (NF-κB) cells inflammatory pathways (metformin) (Wang et al., 2017; Ye et al., 2018). By using senomorphics, researchers aim to turn senescent cells “quiet,” converting them from pro-inflammatory to a more benign state that does not disrupt tissue function. In summary, senolytics and senomorphics offer complementary means to target the senescence component of the senescence–reprogramming axis: senolytics physically remove the source of chronic SASP, whereas senomorphics functionally neutralize the SASP’s harmful effects (Lagoumtzi and Chondrogianni, 2021). Both approaches are moving toward clinical trials for diseases ranging from fibrotic lung disease and osteoarthritis (for senolytics) to Alzheimer’s and cancer (for SASP inhibitors), underscoring their broad therapeutic potential (Cuollo et al., 2020; Liu and Liu, 2020; Ungerleider et al., 2021; Zhao et al., 2025).

**TABLE 1:** Senolytic agents targeting selective elimination of senescent cells.

Agent	Mechanism of Action	Clinical/Preclinical Applications	Reference
Dasatinib	Broad-spectrum tyrosine kinase inhibitor (Src kinases, ephrin signaling, PI3K/AKT pathways)	Human trials (in combination with Quercetin); improved frailty, fibrosis, metabolic function in animal models	PMID: 31542391
Quercetin	Inhibits PI3K/AKT, interferes with BCL-xL pathway	Human trials in combination with Dasatinib; broad-spectrum senolytic activity	PMID: 31542391
Navitoclax (ABT-263)	Selective BCL-2 family protein inhibitor (BCL-2, BCL-xL, BCL-W)	Preclinical studies; *in vivo* clearance of senescent bone marrow and chemotherapy-induced senescent cells	PMID: 26657143
FOXO4-DRI peptide	Disrupts FOXO4-p53 interaction, releasing active p53 to induce apoptosis	Restored fitness and organ function in aged mice	PMID: 35510614
Digoxin, Ouabain	Inhibit Na^+^/K^+^ pumps, disrupt cellular homeostasis	Preclinical models, especially therapy-induced senescence	PMID: 31636264
BPTES	Glutaminase inhibitor, targets altered metabolism in senescent cells	Preclinical	PMID: 36435512
RSL3	Induces ferroptosis in senescent cells	Preclinical	PMID: 35607817
SSK1 (β-gal prodrug)	β-galactosidase activated prodrug, triggers p38 MAPK apoptosis	Preclinical (mouse models)	PMID: 32341413

**TABLE 2:** Senomorphic agents modulating the senescence-associated secretory phenotype (SASP).

Agent	Mechanism of Action	Clinical/Preclinical Applications	Reference
Rapamycin, Everolimus, Temsirolimus	mTOR pathway inhibitors; reduce SASP via NF-κB/STAT3 suppression	Preclinical and early human trials; extend lifespan, reduce inflammaging in animal models	PMID: 23258954
Metformin	AMPK activator; indirectly inhibits mTOR/NF-κB signaling, reduces SASP	Preclinical and human trials (TAME study)	PMID: 35640743
Ruxolitinib	Inhibit cytokine-driven JAK/STAT pathway, block SASP cytokines (IL-6, IL-8)	Preclinical studies; IPF trials showed improved physical function	PMID: 26578790
Parthenolide, BAY 11-7082	Block NF-κB-mediated transcription of SASP components	Preclinical studies; reduced inflammation in mouse models	PMID: 20093358
PTBP1 inhibitors	RNA-splicing factor required for full SASP secretion	Preclinical	PMID: 29990503
PDIA3 inhibitors	Blocks PDIA3, inhibits TGF-β activation and fibrotic SASP	Preclinical	PMID: 32687065 PMID: 36509292
Siltuximab, Canakinumab	Neutralize SASP cytokines directly	Human trials	PMID: 29447987

### 7.2 Partial reprogramming and epigenetic modulators

On the reprogramming side, the challenge is to rejuvenate aged cells or reset malignant cells without causing uncontrolled cell fate reversal or tumorigenesis. Complete reprogramming to pluripotency would erase cellular identity, which is not desirable *in vivo* (Hanna et al., 2010). Instead, controlled partial reprogramming has emerged as a promising strategy (Yoshida, 2015). Partial reprogramming involves brief or attenuated activation of reprogramming factors to rewind some aspects of cellular age while stopping short of full dedifferentiation (Jo et al., 2020). In mouse models of aging, cyclic partial reprogramming (e.g., turning on OSKM for a few days at a time) was sufficient to restore youthful molecular profiles and organ function, while the cells retained their original identity as tissue cells (Yücel and Gladyshev, 2024). Strikingly, tissues like muscle and pancreas in these mice showed improved regeneration, and progeroid mice lived longer without apparent cancer formation (Lavasani et al., 2012). This demonstrates that tissues in an aged animal carry an imprint of youth that can be reawakened (Streit and Xue, 2009). Another remarkable example is ocular gene therapy with partial reprogramming factors: introducing just three Yamanaka factors (Oct4, Sox2, Klf4 – omitting c-Myc) into old mice’s retinal ganglion cells led to restored vision and nerve regeneration (Fang et al., 2013). The aged neurons regained axon growth ability and visual function by regaining a more “youthful” epigenetic state (Yang et al., 2022) (Table 3). These findings show that carefully calibrated reprogramming can reverse functional aspects of aging *in vivo*. To translate this to therapy, scientists are developing inducible systems and transient delivery methods to induce rejuvenation in specific tissues without risking uncontrolled cell proliferation (Tamanini et al., 2018). In addition to genetic factor-based reprogramming, there is growing interest in epigenetic modulators and small molecules that achieve similar rejuvenating effects (Wang et al., 2023). Because the aging process is tightly linked to epigenetic dysregulation, drugs that modify the epigenome can mimic aspects of reprogramming (Pal and Tyler, 2016; Wang et al., 2022). In one study, scientists reported six chemical cocktails capable of reversing cellular aging markers in human cells *in vitro* (Zhang et al., 2022). Within days of treatment, senescent or aged cells treated with these cocktails displayed youthful gene expression patterns and a reset epigenetic “age,” all while maintaining their original cell type identity (Mendelsohn and Larrick, 2019). Such chemical reprogramming strategies aim to achieve safe rejuvenation: resetting the cell’s age and repair capacity without fully stripping its specialized functions (Jiang et al., 2023). Epigenetic drugs already in use (like DNA methyltransferase inhibitors or HDAC inhibitors in cancer therapy) illustrate the feasibility of altering epigenetic states; the new cocktails go further by targeting multiple pathways to induce a concerted age-reversal program (Lakshmaiah et al., 2014). If these findings translate *in vivo*, epigenetic rejuvenation therapy could become a pillar of geriatric medicine–for example, periodic treatments to refresh the epigenome of an aging organ, thereby reducing senescent cell accumulation and improving tissue resilience (Kaur et al., 2022; Haykal et al., 2025). In cancer, epigenetic modulators might coax tumor cells into more differentiated, less aggressive states (a concept known as differentiation therapy, successful in diseases like APL leukemia) (Shekhani et al., 2013). While not traditional “reprogramming” to pluripotency, this approach uses the same principle of reprogramming cell fate–here to push cancer cells out of a stem-like state into a terminal state (like senescence or a post-mitotic differentiation), making them easier to eliminate or control (Aydin and Mazzoni, 2019; Zimmermannova et al., 2021). Together, interventions targeting senescence and those harnessing reprogramming are two sides of a strategy to restore healthy tissue homeostasis (Menendez et al., 2011). Removing or nullifying aberrant senescent cells clears the obstacles to regeneration; inducing partial reprogramming or epigenetic rejuvenation actively promotes the regenerative, youthful program in cells (Rando and Chang, 2012; Cipriano et al., 2024). It is notable that these strategies might be even more powerful in combination. For instance, a transient reprogramming treatment might be paired with senolytics–the reprogramming could rejuvenate the majority of cells, while senolytics eliminate any cells that enter a senescence/SASP state as a side effect, thereby preventing tumorigenic risks (Zhang et al., 2022; Sahu et al., 2024). Although still in early stages, such synergistic therapies exemplify the translational potential of understanding the senescence–reprogramming nexus.

**TABLE 3:** Epigenetic and reprogramming strategies for reversing cellular senescence.

Agent	Mechanism of Action	Clinical/Preclinical Applications	Reference
Partial reprogramming (OSKM factors)	Transient Yamanaka factor (OCT4, SOX2, KLF4, MYC) expression resets epigenetic age	Animal models; improved regeneration, organ function, vision restoration	PMID: 27984723 PMID: 30328784
Decitabine, 5-Azacytidine	DNA methylation inhibitors; reactivate silenced genes	Preclinical studies	PMID: 27039820
Trichostatin A, Butyrate	Increase histone acetylation, restore youthful gene expression patterns	Preclinical longevity studies	PMID: 15695762
Sirtuin activators	Enhance DNA repair, chromatin silencing	Preclinical models, calorie restriction mimetics	PMID: 27028172
SUV39H1, LSD1 inhibitors	Alter histone methylation patterns linked to senescence-associated heterochromatin	Preclinical studies	PMID: 35000672

## 8 Conclusion and future perspectives

This review’s focus on the intersection of cellular senescence and reprogramming reflects a paradigm shift in how we understand aging and cancer biology. Rather than viewing senescence (a hallmark of aging and a tumor suppressor) and reprogramming (a tool for regenerative medicine and a potential tumor risk factor) in isolation, we now appreciate that these processes are deeply interconnected. This crosstalk influences whether tissues degenerate or regenerate, and whether tumors are suppressed or grow aggressively. By emphasizing their interplay, researchers have uncovered novel mechanisms–such as SASP-mediated enhancement of cell plasticity and reprogramming-driven erasure of aging traits–that explain complex phenomena in aging and cancer (from paradoxical pro-regenerative roles of senescent cells to the emergence of cancer stem cell states under stress). The revised evidence base we assembled highlights that the senescence–reprogramming axis is a pivotal regulator of tissue homeostasis, capable of tipping the balance toward repair and renewal or toward pathology.

Looking ahead, leveraging this knowledge requires careful calibration. Therapeutically, we envisage a future where clinicians can modulate cellular identity and senescent cell burden in patients: eliminate harmful senescent cells, reinvigorate aging cells via partial reprogramming, and perhaps even induce senescence in cancers selectively or reprogram cancer cells to a benign state. Achieving this safely is the foremost challenge. Uncontrolled reprogramming carries the danger of teratoma formation or loss of tissue structure, while indiscriminate removal of senescent cells might impair wound healing or tissue integrity. Therefore, precision is key–for example, targeted delivery of reprogramming factors to specific cell types or timed administration of senolytics to avoid interfering with acute injury responses. Advances in gene therapy, senescence biomarkers, and single-cell technologies will aid in this precision. Furthermore, an important future direction is to map the molecular switches between senescence and reprogramming: identifying which transcription factors, cytokines, or metabolic signals determine a cell’s fate towards regeneration versus arrest. Such insights could yield drug targets that tweak this balance without full-blown cellular reprogramming, essentially offering the benefits of rejuvenation with minimal risk.

In conclusion, aligning with the scope of this special issue, we have illustrated that the interplay between cellular senescence and reprogramming is a critical nexus in aging and cancer. By clearly addressing how these processes intersect–co-regulating tissue maintenance, influencing disease progression, and offering dual nodes for therapeutic intervention–we provide a coherent framework that links fundamental cell biology to clinical strategies. Ongoing and future research at this intersection holds the promise of novel therapies: senescence-modulating and reprogramming-based interventions that together could redefine how we treat age-related degeneration and combat cancer. The convergence of these fields exemplifies the adage that in complexity lies opportunity–by understanding the complex crosstalk of senescence and reprogramming, we open up new opportunities to enhance healthspan and combat malignancy with unprecedented sophistication.

## Abbreviations

DDR, DNA damage response; EMT, epithelial–mesenchymal transition; IL-6, interleukin-6; iPSCs, induced pluripotent stem cells; JAK, Janus kinase; KLF4, Kruppel-like factor 4; MYC, MYC proto-oncogene, bHLH transcription factor; mTOR, mechanistic target of rapamycin; NF-κB, nuclear factor kappa-light-chain-enhancer of activated B; OCT4, Octamer-binding transcription factor 4; OIS, oncogene-induced senescence; SASP, senescence-associated secretory phenotype; SOX2, SRY-box transcription factor 2; STAT, signal transducer and activator of transcription.
